# Lung Cancer Metastasis to the Pituitary Gland

**DOI:** 10.7759/cureus.22608

**Published:** 2022-02-25

**Authors:** Joanna Kilbane Myers, Ahmed Abdelrahman, Basil Akpunonu

**Affiliations:** 1 Internal Medicine, University of Toledo, Toledo, USA; 2 Nuclear Medicine, Icahn School of Medicine at Mount Sinai, New York, USA

**Keywords:** pituitary metastases, metastasis to the pituitary, primary lung carcinoma, pituitary metastasis, pituitary mri, clinical endocrinology

## Abstract

Common sites of lung cancer metastasis include the bone, brain, liver, and adrenal gland. Cancer metastasis to the pituitary gland or sellar region is a rare finding. Here, we present a case of pituitary gland metastasis from underlying lung cancer in a patient presenting with a predominance of pituitary symptoms over respiratory symptoms. A 48-year-old female was admitted to the hospital with progressive visual deficits, intractable headaches, constant nausea and vomiting, fatigue, polyuria, and polydipsia for about three months, all consistent with pituitary symptoms associated with secondary adrenal insufficiency, secondary hypothyroidism, and central diabetes insipidus.

A brain MRI done two months earlier revealed a large mass in the pituitary gland and sella turcica area. Biochemical test abnormalities consistent with pituitary hormonal insufficiencies were noted, and subsequent imaging showed an enlarging pituitary mass and extensive metastases to the bones, brain, liver, adrenal gland, and lymph nodes. Bone biopsy was consistent with poorly differentiated adenocarcinoma of the lung as the primary site. The young age of this patient is uncommon compared to most patients with pituitary metastasis. Worsening pituitary symptoms with an enlarging pituitary mass and widespread metastases should alert consideration for pituitary metastasis and a search for a primary cancer site. Pituitary metastasis portrays a poor prognosis.

## Introduction

Lung cancer most commonly metastasizes to the bone, liver, nervous system, and adrenal gland [1]. Cancer metastasis to the pituitary gland or sellar region is a rare finding, encompassing less than 1% of intracranial metastatic lesions [2]. The diagnosis of metastasis to the pituitary is often made on autopsy/postmortem examinations, diagnostic biopsies, surgical resection, and debulking procedures [2]. Pituitary adenomas are slow-growing, while pituitary metastases are fast-growing and produce more profound symptoms [2-4].

Small lesions (<10 mm) are usually functional and produce increased hormones, while large lesions (>10 mm) usually do not secrete hormones, and patients with large lesions suffer from the mass effect of the lesions, causing hypothyroidism, hypocortisolism, hypogonadism, and central diabetes insipidus marked by polyuria and polydipsia, which have been regarded as the hallmarks of pituitary metastasis [5]. Symptoms suggestive of pituitary gland involvement, therefore, include headaches, visual problems, nausea and vomiting, changes in behavior, fatigue, weight loss, muscle weakness, generalized malaise, and sexual dysfunction.

Advanced diagnostic radiological modalities such as computed tomography (CT) scans and positron emission tomography (PET) scans aid in the diagnosis and obviate the need for pituitary biopsy when primary or extensive metastatic lesions are known to exist. Features that radiologically suggest metastasis to the pituitary gland and help distinguish from pituitary adenoma include widespread metastatic disease throughout the rest of the body, relatively normal size of the pituitary fossa that suggests growth in a short period, bone deconstruction rather than remodeling, dural thickening, irregular edges, and dumbbell shape of the pituitary gland as the sellar diaphragm has not had time to be stretched [2].

The most common primary neoplasms that cause pituitary metastasis are breast and lung cancer [2]. We present a case of a 48-year-old female presenting with a rapidly growing pituitary mass and pituitary symptoms. She was noted to have widespread metastasis to the bone, adrenal gland, brain, liver, and pituitary, with the lung being the primary site. The pituitary metastasis side effects were her predominant symptoms. However, patients are most often asymptomatic from pituitary metastasis [2]. Pituitary metastasis is a reflection of advanced and aggressive disease with late diagnosis and poor prognosis. It should be distinguished from pituitary adenomas. Due to the patient’s young age compared to most patients with pituitary metastases, our case aims to highlight the importance of including pituitary metastasis in the differential diagnosis in middle-aged patients with a pituitary tumor, diabetes insipidus, and ophthalmoplegia.

## Case presentation

A 48-year-old white female with a 36 pack-year smoking history presented to the hospital with chief complaints of worsening fatigue, intractable headaches, and blurred vision over the past three months. Associated symptoms included daily nausea, progressive anorexia with 25-lb weight loss, lightheadedness, exertional shortness of breath, cold intolerance, hair loss, dry skin, polyuria, polydipsia, abdominal pain, and diarrhea. She smoked one pack of cigarettes daily since the age of 12, and she did not drink alcohol. Mother died of lung cancer at age 58.

Two months prior to her presentation, the patient underwent brain MRI for persistent headaches and vision changes, which revealed an enlarged pituitary measuring 1.5 cm in axial view × 1.3 cm in sagittal view. The patient was instructed to follow up with an endocrinologist, but because of COVID-19, she was unable to secure a timely appointment.

On physical examination, she was alert and oriented and in moderate distress, complaining of generalized aches, bone pain, and difficulty with her vision. Vital signs included BP of 85/69 mmHg, pulse of 65 beats per minute, respiratory rate of 13 breaths per minute, and temperature of 98.2°F. No skin lesions or lymphadenopathy were noted. Lungs were clear and without adventitious sounds. The patient had no jugular venous distention or hepatojugular reflux. The abdomen was soft with normal bowel sounds, and there was no hepatosplenomegaly. Her paraspinal muscles were tender to palpation and percussion of the thoracolumbar vertebrae. Her myoneurological examination was limited by extreme discomfort at all times as she felt better with both eyes closed. She maintained central vision but was unable to see well peripherally.

**Figure 1:**
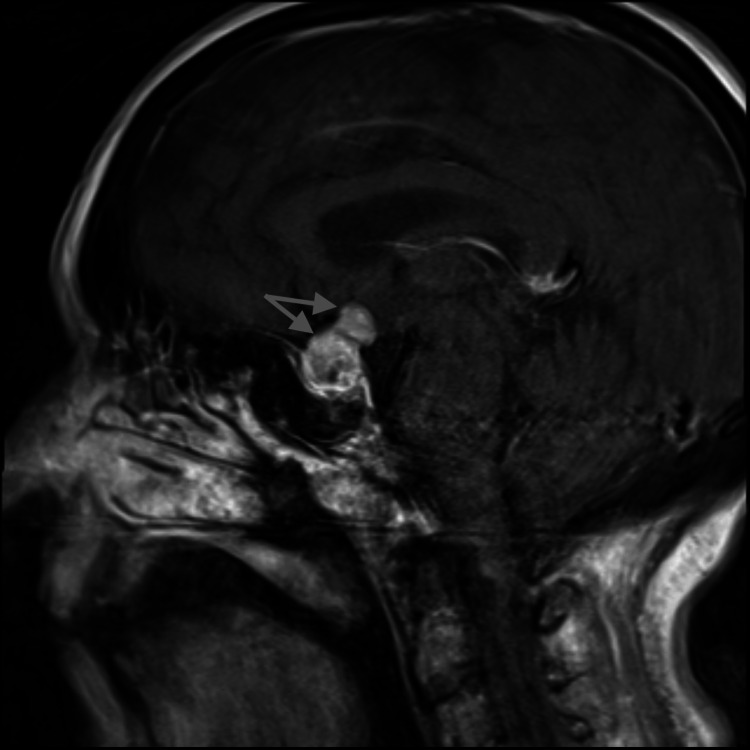
T1 post contrast brain MRI with pituitary protocol (sagittal image) Image showing a dumbbell lesion in the pituitary consistent with metastatic disease to the pituitary Gray arrows: pituitary mass

**Figure 2:**
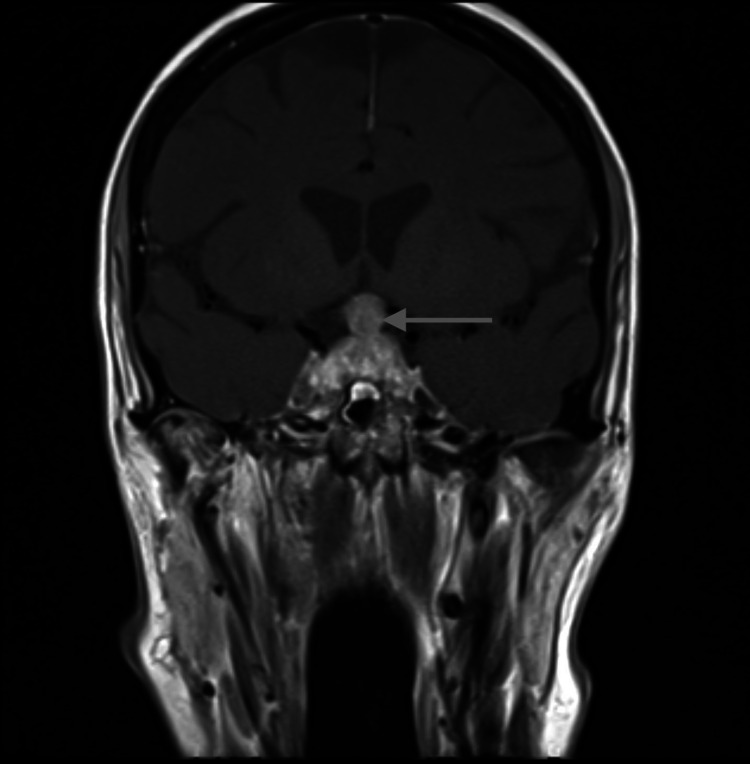
T1 post contrast brain MRI with pituitary protocol (coronal image) Image showing the mass effect on the optic chiasm and optic nerves Gray arrow: pituitary mass

A repeat brain MRI with pituitary protocol was done because of worsening headaches and demonstrated significant interval growth of the pituitary lesion now measuring 2.4 cm × 1.6 cm × 1.9 cm in greatest dimension, now extending into the suprasellar cistern causing mass effect on the optic chiasm and optic nerves (Figures 1, 2).

A new 5-mm enhancing lesion on the MRI in the right cerebellar hemisphere with surrounding edema suggested a metastatic focus. Given the presence of the rapidly growing pituitary lesion and the new cerebellar lesion, the possibility of metastatic disease from an unknown primary malignancy was considered. Subsequently, a chest CT with contrast showed a macrolobulated mass in the left upper lobe of the lung with extension to the lateral pleural surface measuring 2.5 cm × 2.4 cm × 2.3 cm. MRI of the thoracic and lumbar spine with contrast revealed multifocal lytic lesions throughout the thoracic and upper lumbar spine, with very large lesions at T1 and T3. Computed tomography of the abdomen and pelvis with contrast also showed multiple lesions in the liver, small lytic lesions in the bones including the proximal left femur, both iliac bones, and several lumbar and thoracic vertebral bodies, and a left adrenal gland mass measuring 3.4 cm × 1.6 cm, consistent with metastatic disease. A CT-guided biopsy of the right iliac bone lesion revealed metastatic poorly differentiated epithelial malignancy.

Immunostaining of the bone lesion was performed with adequate controls. The results for the following stains were negative: MART-1, S100, GATA3, SOX10, p40, TTF1, napsin, CK20, GCDFP-15, ER, PR, PAX8, WT1, p63, synaptophysin, chromogranin, HepPAR1, podoplanin, and p16. The differential diagnosis here still includes primary lung neoplasm, and given the radiological findings, adenocarcinoma of the lung is the favored primary diagnosis after immunohistochemical and morphologic workup. The immunohistochemical profile effectively ruled out with significant confidence the following primary malignancies: breast, ovarian, liver, adrenal, kidney, bladder, colon, uterine, cervical, neuroendocrine, and melanoma. The patient did not consent to lung biopsy because of concern for pneumothorax complications and its treatments.

Values with low cortisol, adrenocorticotropic hormone (ACTH), free thyroxine (T4), triiodothyronine (T3), thyroid-stimulating hormone (TSH), follicular-stimulating hormone (FSH), and luteinizing hormone (LH) consistent with central hypocortisolism, secondary hypothyroidism, and decreased gonadotropin levels are presented in Table 1. Values of increased serum sodium and serum osmolality and low urine osmolality consistent with diabetes insipidus are also presented in Table 1.

**Table 1:** Endocrinological evaluation TSH: thyroid-stimulating hormone; free T4: free thyroxine; free T3: free triiodothyronine; ACTH: adrenocorticotropic hormone; DHEA sulfate: dehydroepiandrosterone sulfate; FSH: follicle-stimulating hormone; LH: luteinizing hormone

Hormone/electrolyte/osmolality	Levels at admission	Levels at discharge	Normal levels
TSH	0.5 uIU/mL		0.49–4.67 uIU/mL
Free T4	0.52 ng/dL	0.77 ng/dL	0.61–1.60 ng/dL
Free T3	1.70 pg/mL		2.50–3.90 pg/mL
Free cortisol	0.13 ug/dL		0.10–0.63 ug/dL
ACTH	1.4 pg/mL		7.2–63.3 pg/mL
Glucose	53 mg/dL	101 mg/dL	65–99 mg/dL
Aldosterone	5.6 ng/dL		3.1–35.4 ng/dL
DHEA sulfate	3 ug/dL		19–231 ug/dL
FSH	1.3 mIU/mL		16.7–113.6 mIU/mL
LH	<0.2 mIU/mL		10.9–58.6 mIU/mL
Serum sodium	156 mmol/L	151 mmol/L	134–146 mmol/L
Serum osmolality	326 mOsm/kg		280–300 mOsm/kg
Urine osmolality	81 mOsm/kg		300–1,300 mOsm/kg

Further diagnostic studies and therapeutic interventions including vision-sparing procedures and loading or stimulating tests were discussed with the patient and family, and they decided against any further interventions and elected a palliative/hospice care approach because of the poor prognosis.

## Discussion

Metastatic disease to the pituitary gland is rare. While pituitary adenomas comprise 75% of all tumors of the sellar region [4], pituitary metastases make up less than 1% of intracranial metastatic lesions [2]. Upon postmortem examination, between 0.14% and 28.1% of patients with brain metastasis were found to have pituitary metastasis [5]. The percentage of living patients who present with pituitary symptoms due to metastatic disease is even less.

In a retrospective study of patients with pituitary metastases from 1996 to 2015, the breast and lung were the most frequent sites of primary malignancy overall [4]. In women, the most common primary site is the breast. In a case report and literature review in 2004, breast cancer was the primary site for pituitary metastasis in 39.7% of patients, and lung cancer comprised 23.7% of cases [5].

Most patients with pituitary lesions present asymptomatically; however, symptoms of hypopituitarism are the presenting complaint in 20%-30% of patients with pituitary metastasis from underlying malignancy [2]. Headache, visual problems, nausea and vomiting, change in behavior, fatigue, weight loss, muscle weakness, sexual dysfunction, and diabetes insipidus marked by polyuria and polydipsia are the most commonly reported symptoms in pituitary metastasis [2]. Our patient’s predominant symptoms were central diabetes insipidus, secondary adrenal insufficiency, and secondary hypothyroidism. While diabetes insipidus may present due to a pituitary adenoma, only 1% of pituitary adenomas will cause diabetes insipidus [5]. Pituitary metastasis causing central diabetes insipidus was found in 3.27% of patients with primary lung cancer, with increased incidence compared to central diabetes insipidus in patients with pituitary adenomas [6]. Approximately half of the patients with pituitary metastasis from all primary locations present with diabetes insipidus [2], further indicating that this patient’s enlarging pituitary was due to metastatic disease.

The proposed mechanisms for pituitary metastasis include direct hematological spread, spread through the hypothalamic portal vessels, meningeal spread via the suprasellar cistern, and skull base metastasis [5]. The posterior pituitary is particularly susceptible to metastatic spread due to blood supply from the systemic circulation via hypophyseal arteries in contrast to the anterior pituitary, which is supplied through the portal circulation [5].

Radiographic features that are now accepted to suggest pituitary metastasis and distinguish from pituitary adenoma include the relative normal size of the pituitary fossa with mass suggesting rapid growth over a short period of time, bone destruction rather than remodeling, irregular edges, widespread metastasis to other organs including the brain, and dumbbell shape of the pituitary as the sellar diaphragm has not had time to stretch [2].

The average age of patients diagnosed with pituitary metastasis is 58.6±7.8 years [6]. Previous case reports and studies have outlined the importance of including pituitary metastasis in the differential diagnosis in a patient aged 50 or older, with a rapidly growing pituitary tumor, and features of diabetes insipidus and ophthalmoplegia. We agree that it should be extended as well to those with widespread metastatic disease. We aim to acknowledge this recommendation in even younger patients in their 40s due to our finding in a 48-year-old patient.

The treatment options for metastatic pituitary disease include symptomatic treatment with hormone replacement and a combination of surgery, chemotherapy, and/or radiation [4]. Hormone replacement including levothyroxine, hydrocortisone steroids, and/or desmopressin improves symptoms and quality of life. Surgical resection of pituitary metastasis is difficult but may be pursued if symptoms are severe or if the pituitary is rapidly expanding in size. Due to poor prognosis and limited survival times, incorporating palliative care is important in the management of pituitary metastasis due to advanced disease at the time of diagnosis.

Patients with metastasis to the pituitary gland were found to have a poor prognosis, depending upon the primary site of malignancy and the degree of metastasis. Patients with pituitary metastasis had a median length of survival of 16.5 months (95% confidence interval: 10.7-25.4) [4]. Patients with surgical resection may have increased lengths of survival, which may contribute toward lengthening survival times in pituitary metastasis in recent years [4]. One patient with symptomatic pituitary metastasis from primary lung cancer in a retrospective study was still alive three years after diagnosis, following intravenous chemotherapy and radiation to the sellar region [6]. Our patient elected palliative/hospice care.

## Conclusions

Pituitary metastasis diagnoses are often made late because of vague symptoms. A rapid pituitary gland enlargement with severe pituitary symptoms should enable consideration for metastatic disease rather than a pituitary adenoma. Symptoms outside the scope of panhypopituitarism, including shortness of breath, cachexia, and diarrhea, should further raise suspicion for metastatic disease rather than a pituitary adenoma. More studies should examine metastasis to the pituitary and hypothalamic area in general due to low prevalence and minimal literature review regarding this location of metastasis.
